# Vertigo

**Published:** 1883-08

**Authors:** 


### SELECTIONS.

### Vertigo.

In the beginning of a communication on this subject by Dr. Edward Woakes, in the *Brit. Med. Jour.*, April 28, 1883, the author says:

The point of view which I propose to submit for consideration in this paper is, that all vertigo is essentially auditory in its seat; that is to say, whenever the symptom of giddiness, disturbance of the equilibrium, defective gait, falling, or whatever modification of these may characterize the symptom of vertigo, its immediate occurrence is due to a modification of tension of the part of the labyrinthine fluid contained in the semicircular canals, and the appreciation by the ampullar nerve-apparatus of this change of tension.

